# Intelligent Evaluation and Dynamic Prediction of Oysters Freshness with Electronic Nose Non-Destructive Monitoring and Machine Learning

**DOI:** 10.3390/bios14100502

**Published:** 2024-10-14

**Authors:** Baichuan Wang, Yueyue Li, Kang Liu, Guangfen Wei, Aixiang He, Weifu Kong, Xiaoshuan Zhang

**Affiliations:** 1 Beijing Laboratory of Food Quality and Safety, College of Engineering, China Agricultural University, Beijing 100083, China; b20193070600@cau.edu.cn (B.W.); 15856916264@163.com (K.L.); 2 Yantai Institute, China Agricultural University, Yantai 264670, China; 3 School of Information and Electronic Engineering, Shandong Technology and Business University, Yantai 264005, China; guangfen.wei@sdtbu.edu.cn (G.W.);

**Keywords:** oyster freshness, intelligent evaluation, GA-BP, electronic nose, gas sensor, non-destructive monitoring

## Abstract

Physiological and environmental fluctuations in the oyster cold chain can lead to quality deterioration, highlighting the importance of monitoring and evaluating oyster freshness. In this study, an electronic nose was developed using ten partially selective metal oxide-based gas sensors for rapid freshness assessment. Simultaneous analyses, including GC-MS, TVBN, microorganism, texture, and sensory evaluations, were conducted to assess the quality status of oysters. Real-time electronic nose measurements were taken at various storage temperatures (4 °C, 12 °C, 20 °C, 28 °C) to thoroughly investigate quality changes under different storage conditions. Principal component analysis was utilized to reduce the 10-dimensional vectors to 3-dimensional vectors, enabling the clustering of samples into fresh, sub-fresh, and decayed categories. A GA-BP neural network model based on these three classes achieved a test data accuracy rate exceeding 93%. Expert input was solicited for performance analysis and optimization suggestions enhanced the efficiency and applicability of the established prediction system. The results demonstrate that combining an electronic nose with quality indices is an effective approach for diagnosing oyster spoilage and mitigating quality and safety risks in the oyster industry.

## 1. Introduction

Oysters, a highly prized type of shellfish, are widely recognized for their medicinal and culinary value [1,2,3]. However, the common practice of shelling oysters prior to market sale exposes them to various issues, including belly breakage, microbial contamination, discoloration, and spoilage [4]. Consequently, it is crucial to accurately monitor, assess, and predict the freshness of oysters throughout their transportation, storage, processing, and marketing.

Conventional evaluation methods, such as sensory assessment [5], physical and chemical analysis [6], and microbial detection [7], have inherent limitations. Kuuliala et al. [8] identified and qualified volatile organic compounds through microbiological, chemical, and sensory analyses over storage periods at 4 °C and 8 °C to assess spoilage in Atlantic cod. To measure the odors of raw shrimp, Luzuriaga et al. [9] developed an electronic nose equipped with 12 conduction polymer sensors, achieving a classification accuracy of 99.2% for sulfite-treated shrimp. These traditional methods often depend on subjective judgment, involve complex procedures, and require lengthy detection cycles, which may negatively affect oysters. In contrast, non-destructive monitoring [10] presents clear advantages by accurately identifying nutrients and harmful substances in food while efficiently classifying food quality. Currently, the leading non-destructive monitoring methods include Fourier transform infrared spectroscopy (FTIR) [11], computer vision analysis [12], ultrasonic detection [13], and electronic nose technology [14].

As a monitoring method, the electronic nose can comprehensively capture and analyze the overall characteristics and potential attributes of the tested sample by constructing a sensor array with specific recognition capabilities and an advanced pattern recognition system. It has been widely utilized in various fields, including medical diagnosis [15], environmental monitoring [16], food quality analysis [17], and the tobacco industry [18]. However, the limited odor recognition ability of the electronic nose, combined with fluctuating external environmental conditions and the complex and diverse characteristics of oysters, presents challenges in achieving a comprehensive evaluation of oyster quality and accurately predicting its freshness solely through monitoring changes in the odor and environmental conditions.

Machine learning algorithms [19], such as artificial neural networks (ANNS), support vector machines (SVMS), and random forests, have been integrated with electronic noses to the enhance the accuracy and reliability of odor detection and classification. Harsono et al. [20] presented an electronic nose to detect several types of coffee and compared Logistic Regression (LR), Linear Discriminant Analysis (LDA) and K-nearest Neighbors (KNN) classification methods. The KNN method showed better performance with an accuracy value of 97.7%. Huang et al. [21] designed a portable, battery-powered electronic nose system for odor characterization and used machine learning such as SVMS to classify the VOCS based on the dataset result. The sensitivity and specificity were 98.5% and 98.6% for the wine test. Therefore, we also employed the Internet of Things (IoT) combined with machine learning modeling, which not only enabled the real-time dynamic monitoring of the aquatic products but also enhanced the accuracy in quality assessment and freshness prediction. This approach possesses strong multi-parameter sensing capabilities along with self-learning and adaptive adjustment abilities. Commonly used machine learning algorithms include decision trees, random forests, support vector machines, artificial neural networks, etc. [22]. The detection of lung cancer using an electronic nose is achieved through a novel ensemble learning framework. By inputting multidimensional information into this algorithm, it has been successfully validated for comprehensive evaluation and freshness prediction in foods such as meat [23], fish [24], fishmeal [25], shrimp [26], and oysters [27].

Based on previous research, this paper proposes a machine learning-based model for predicting oyster freshness. The model employs electronic nose technology to monitor the volatile gasses emitted by oysters in real-time and intelligently integrates multiple quality indicators to create a comprehensive system for assessing oyster quality. In this study, the original gas data are normalized and subjected to dimensionality reduction using principal component analysis. The neural network freshness prediction model is optimized through a genetic algorithm (GA) [28] and considers the overall impact of environmental factors and quality indicators on oyster shelf life [29]. This work offers valuable insights into shellfish product quality assessment and freshness prediction.

## 2. Materials and Methods

### 2.1. Mechanism Analysis and Model Framework

**Figure 1:**
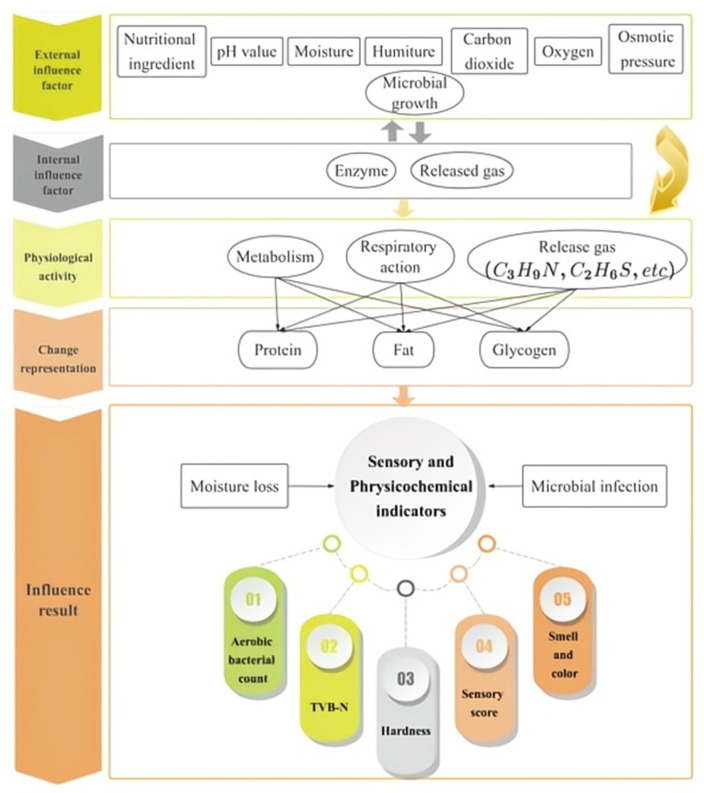
Fresh oyster quality change mechanism during the preservation process.

The mechanism of oyster quality changes during storage and transportation is depicted in Figure 1. Fluctuations in temperature and humidity throughout production, processing, distribution, and sales accelerate the growth and activity of microorganisms and spoilage bacteria, leading to nutrient decomposition and the production of toxic substances, ultimately resulting in oyster deterioration. Additionally, changes in external environmental factors, such as nutrient composition, pH value, moisture content, carbon dioxide content, oxygen content, and osmotic pressure, also impact the physiological, biochemical, and enzymatic reactions of oysters [30]. On one hand, the presence of fatty acids in oysters and the breakdown of proteins result in the production of spoilage compounds, such as amino acids, peptides, and volatile gasses [31] (including trimethylamine [32], ammonia [33], and dimethyl sulfide [34]), exacerbating the deterioration of oysters. On the other hand, the spoilage compounds generated by oysters themselves respond to external factors, such as increased microbial metabolism and decreased oxygen levels, which lead to heightened anaerobic respiration. Consequently, adverse changes in external environmental conditions further accelerate the degradation of oysters, presenting greater challenges for maintaining their freshness and ensuring food safety.

The assessment and prediction of oyster freshness result from the complex interaction of numerous factors. This process is influenced not only by environmental conditions that promote quality indicators, but also by the reciprocal effects of these quality indicators on environmental factors. By comprehensively considering the real-time monitoring of temperature, humidity, gas parameters, and physical indicators such as hardness, color, and odor, as well as chemical indicators like pH and TVB-N, and biological changes including microbial colonies, we can observe their correlations that demonstrate fluctuations in oyster quality during storage and transportation. This holistic approach facilitates a more accurate prediction and evaluation of the trends in oyster freshness.

### 2.2. System Architecture

**Figure 2:**
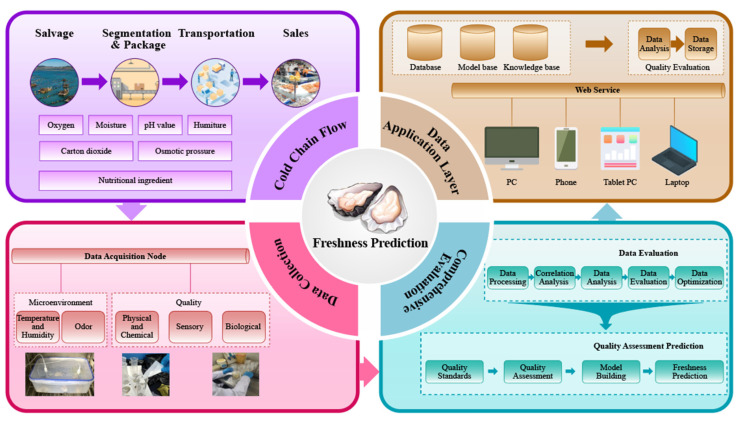
Comprehensive framework for monitoring, traceability, and freshness prediction in the oyster cold chain system.

In conjunction with the characteristics of the oyster transportation process, this study established an experimental scheme for non-destructive monitoring and multi-parameter detection (Figure 2). A real-time monitoring system was developed to facilitate the continuous monitoring of the microenvironment signals of oysters during storage, utilizing the assessment and prediction capabilities of an electronic nose. The system primarily consists of four modules: the process analysis layer, the data acquisition layer, the comprehensive evaluation layer, and the data application layer.

**Process analysis layer:** Throughout the transportation, storage, processing, and sale of oysters, challenges such as belly rupture, microbial growth, and deterioration are likely to arise. Therefore, it is essential to perform data collection, system assessment, freshness prediction, and data display for oysters across these processes;

**Data collection layer:** In accordance with the evaluation index and the requirements outlined by the process analysis module, the data collection module gathers various key parameters, including odor information, the total number of colonies, TVBN, hardness, the microbial index, and the sensory index. These data are acquired through sensors, as well as physical, chemical, microbial, and sensory experiments. After undergoing analog-to-digital conversion, the collected data are transmitted to the evaluation module via the transmission module for further formalization and quantitative processing;

**Comprehensive evaluation layer**: Develop a predictive model for the analysis and optimization of the gathered data. Identify quality indicators and environmental signals that are highly relevant for subsequent evaluation and prediction modeling. Perform the statistical analysis and assessment of the key collected data, and select significant indicators to establish evaluation criteria, collectively forming the predictive model;

**Data application layer**: The data are stored in the sending database, while the modeling results are housed in the model library. The prediction results are transmitted to the knowledge base for storage, and communication with the mobile terminal is facilitated through the mobile communication switching center.

### 2.3. Sample Collection and Processing

Before commencing each experiment, large quantities of alive oysters of similar sizes are to be procured from the breeding farm located in Yantai, Shandong. After live transport to the laboratory, the shells will be removed using specialized tools. The initial step involves selecting oyster meat of comparable shapes and sizes, with each piece weighing approximately 20 g, to conduct a series of experiments. Subsequently, 11 pieces of oyster meat, totaling about 220 g, will be collected for the electronic nose experiment at a specified temperature. Furthermore, in order to enhance the effectiveness and accuracy of the assessment results, six different sets of samples are taken for the same analysis experiments at each different temperature, respectively. The mean data from the six trials are considered for decision making.

### 2.4. Oyster Nose Design and Measurements

**Figure 3:**
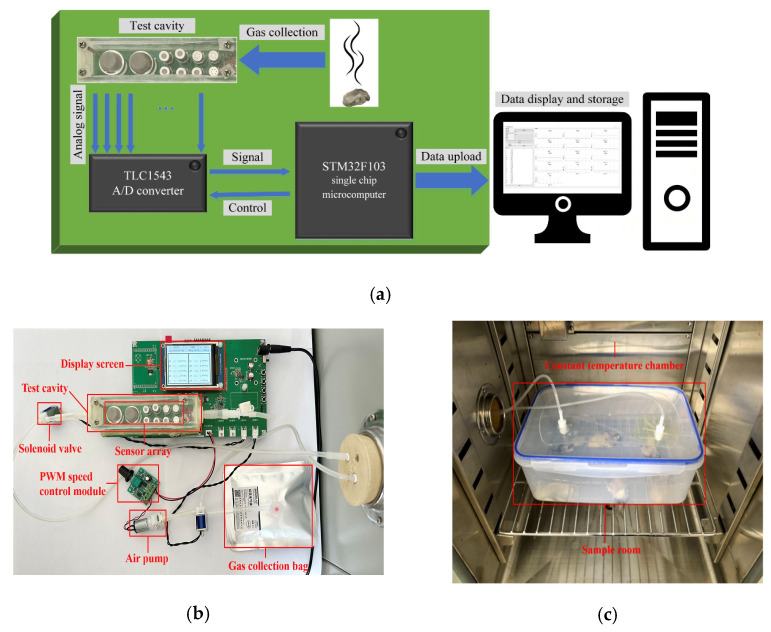
Architecture of the electronic nose monitoring equipment. (**a**) Schematic representation of the testing system; (**b**) actual images of the odor monitoring system; (**c**) test chamber with constant temperature.

The datasets utilized in this study were obtained from the independently developed odor test system designed by the laboratory, as illustrated in Figure 3a. The hardware of the system is centered around the STM32F103 single-chip microcontroller, (STMicroelectronics, Geneva, Switzerland) which serves as its core controller [35]. Analog signal acquisition is conducted using the multi-channel 12-bit analog-to-digital converter TLC1543 (Texas Instruments, Dallas, TX, USA), which provides high resolution, fast speed, and seamless integration with microcontrollers [36]. Furthermore, an LCD screen is incorporated into the system to display real-time sensor voltage readings, as well as temperature and humidity levels within the test chamber, along with the operational status of the solenoid valve and air pump. This feature enhances the visual observation of experimental conditions for test personnel.

The odor test system is illustrated in Figure 3b, which demonstrates the integration of gas collection equipment, including air pumps and various hardware components, into the system’s printed circuit board (PCB). The test system is connected to a constant temperature test chamber (Figure 3c) via a food-grade rubber tube, which contains the test sample. Once the sampled gas enters the test chamber, it elicits a response from the sensor array before being returned to the chamber through a hose.

In accordance with the sensor characteristics and the variations in volatile gasses during the refrigeration of oysters, a sensor array comprising ten distinct MOS sensors with varying sensitivity profiles was selected. When the target gas interacts with the gas-sensitive material of the MOS sensor, a physical and chemical reaction occurs, resulting in a change in the resistance within the gas-sensitive material. The type or concentration of the detected gas can be determined by measuring the voltage signal change that corresponds to this resistance alteration. The models of each sensor and their respective measurable gasses are detailed in Table 1. Furthermore, digital humidity and temperature sensors were incorporated into the prototype alongside the gas sensors to monitor fluctuations in relative humidity and temperature.

**Figure 4:**
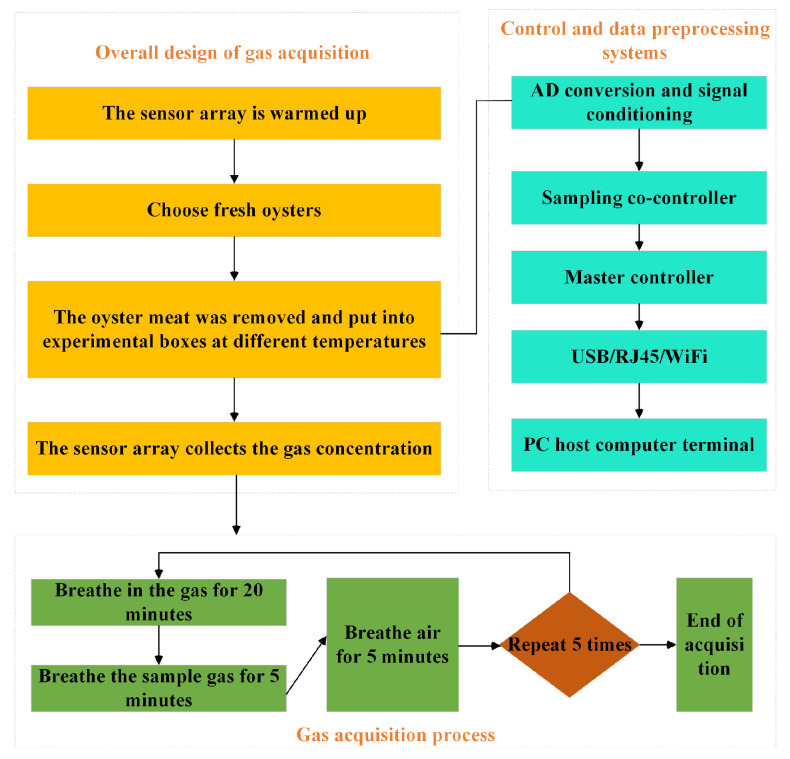
Testing for the volatile gas components of oysters.

Oysters were continuously monitored in a controlled gas flow environment, with each cycle comprising 20 min of thorough cleaning followed by five collection events. Each collection event included a 5 min sampling period and a brief cleaning interval, with a sampling frequency of 1 Hz. The individual sample path and cleaning circuit are regulated by the gas path selection module on the testing equipment, as illustrated in Figure 4. The experimental procedure for oyster samples is outlined as follows:

**Step 1:** Connect the sensor array to the test system and preheat the sensors for one week;
**Step 2:** Place 11 oysters in an enclosed detection gas chamber within a modified polypropylene fresh-keeping box (30 cm × 20 cm × 12 cm), which is then placed in a constant temperature and humidity chamber set at temperatures of 4 °C, 12 °C, 20 °C, and 28 °C, respectively;
**Step 3:** Connect the constant temperature and humidity box to the test system, open the airflow control switch, and initiate data collection using upper computer software until complete decay of the sample;
**Step 4:** Conclude the experiment by organizing obtained datasets into classified labels.

### 2.5. GC-MS Analysis of Volatile Compounds

To gain deeper insights into the chemical composition underlying sensory variation, volatile compounds from oysters stored at 28 °C for 48 h were subjected to GC–MS analysis. The GC–MS system utilized standard laboratory instrumentation and reagents commonly employed in analytical laboratories, including a triple quadrupole tandem mass spectrometer (GC-2030/GCMS-TQ8040NX, Shimadzu Technologies, Kyoto, Japan) equipped with a CP-Sil 8CB column (50 m length, 0.32 mm inner diameter, 5.0 μm film thickness, Agilent Technologies, Utrecht, The Netherlands), methanol (chromatographic grade, Dikma Technologies, Shanghai, China), and a methyl sulfide standard (Macklin Technologies, Twain Harte, CA, USA). A gas bag was used to collect the gas monitored by the electronic nose for direct GC-MS detection.

The pretreatment process was conducted as follows: (a) 100 mL of volatile gas was collected at 28 °C for 48 h directly using a gas collecting bag from the gas chamber of the electronic nose, and (b) 1 mL of the collected gas was extracted with an injection needle for GC–MS detection.

The GC parameters were as follows: helium served as the carrier gas at a flow rate of 4 mL/min. The initial temperature of the chromatographic column was set at 50 °C and maintained for 4 min, after which it was increased to 150 °C at a rate of 20 °C/min, held for 5 min, and then further increased to 250 °C at a rate of 40 °C/min. The mass spectrometry (MS) conditions were as follows: electron-impact mass spectra were generated at 70 eV with a mass-to-charge (*m*/*z*) scan range of 33–325 (0.3 s interval). The ion source temperature was set to 200 °C, and the detector interface temperature was set to 300 °C. Solvent elution was delayed by 2.2 min. All the measurements were performed in triplicate. For positive identifications, the retention indices and mass spectra of the unknowns were compared with those of an authentic methyl sulfide standard substance analyzed under identical conditions.

### 2.6. Evaluation Index

The TVBN, microbial analysis, sensory evaluation, and texture analysis were chosen for a comprehensive assessment of oyster freshness changes. Six oyster samples (approximately 160 g) were selected for the TVBN value, the total number of colonies, and sensory evaluation. Three oyster samples (approximately 80 g) were chosen for texture analysis. The specific time points of sampling during the experiment are presented in Table 2.

#### 2.6.1. TVBN

Volatile basic nitrogen, which includes alkaline nitrogenous compounds such as ammonia and amines that result from the degradation of protein in meat [37], serves as a crucial indicator for evaluating the freshness of aquatic products. This is due to their high protein and amino acid content, which undergoes enzymatic and bacterial decomposition, leading to the generation of alkaline nitrogenous substances during spoilage.

The TVB-N experiment procedure is as follows:

**Step 1:** Firstly, weigh 1 g of magnesium oxide for later use;
**Step 2:** Next, grind the oyster sample and weigh the resulting 10 g oyster sample into the distillation tube;
**Step 3:** Then, add 75 mL water to the distillation tube containing the oyster sample and shake until evenly dispersed in the solution, allowing it to impregnate for 30 min;
**Step 4:** After standing, add 1 g of magnesium oxide to the distillation tube;
**Step 5:** The instrument setup involves using a volume of alkali and diluted water at 0 mL and a receiving solution of boric acid at 30 mL;
**Step 6:** Finally, computer measurement begins after setting up the instrument; start distillation with a duration of three min.

The following classification grades for freshness were established: TVBN content of less than 15 mg/100 g for fresh, 15–25 mg/100 g for moderately fresh, and greater than 25 mg/100 g for spoilage.

#### 2.6.2. Microbiological Analysis

The freshness of oysters can be assessed through microbiological inspection, as they are particularly vulnerable to contamination by microorganisms during production, transportation, and storage.

Based on experimental data and in accordance with GB 4789.2-2016 “National Standard for Food Safety: Determination of the Total Number of Colonies for Microbiology Inspection of Food” [38], this study categorizes oyster freshness levels as follows: samples with a total colony count of less than 6.70 log CFU/g are deemed fresh, samples ranging from 6.70 to 7.70 log CFU/g are classified as sub-fresh, and samples exceeding 7.70 log CFU/g are considered rotten.

#### 2.6.3. Sensory Analysis

Five carefully selected, professionally trained and experienced assessors were chosen to conduct a comprehensive evaluation of the color, aroma, and texture of the oysters. Based on the average final scores, the oysters were categorized as fresh, sub-fresh, or spoiled. Specifically, an average score of 8 or above indicates freshness, a score between 5 and 8 indicates sub-freshness, and any other score indicates spoilage. The specific evaluation criteria are detailed in Table 3.

#### 2.6.4. Texture Analysis

The texture analysis was performed using the Brookfield AMETEK Texture Analyzer (Texture Pro 1.0.19 Advanced Edition). Measurements were taken from the adductor muscle of the oyster, deliberately avoiding internal organs and marginal areas. The data analysis software, Texture Pro 1.0.19, which is connected to the texture analyzer, was employed to obtain hardness parameter values and facilitate data processing.

#### 2.6.5. Statistics Analysis

After processing each independent sample, a parameter analysis was conducted to further investigate the impact of processing and storage time on specific parameters. A two-factor analysis of variance (ANOVA) was performed using SPSS software (International Business Machines Corporation, version 20, New York, NY, USA) to examine these effects. The Pearson correlation coefficient was utilized to explore potential associations between the parameters. Additionally, Duncan’s multivariate range test was employed to identify significant differences between means at the *p* ≤ 0.05 level.

### 2.7. Evaluation and Prediction Models

**Figure 5:**
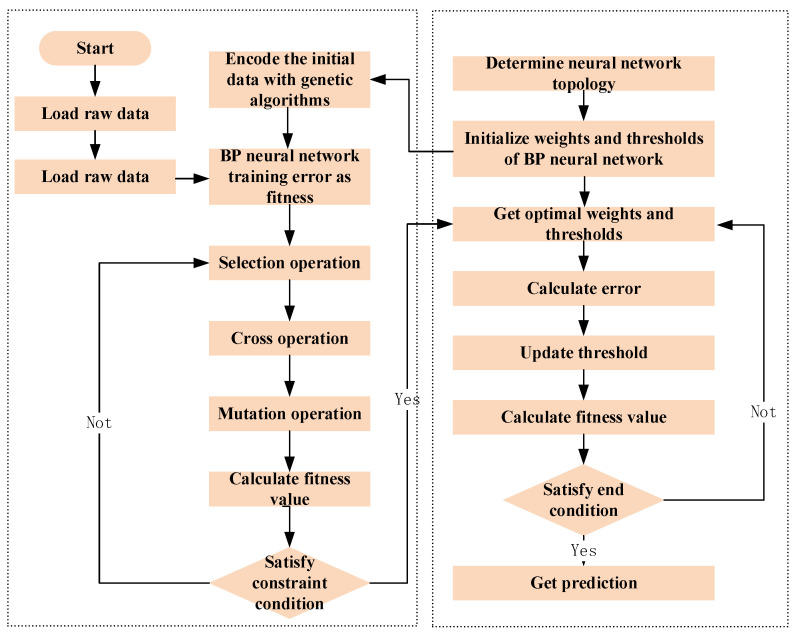
Gas quality model modeling flow chart.

The supply chain comprises numerous oysters, and monitoring volatile gas components individually with a gas sensor array would necessitate considerable time and resources. To elucidate the relationship between environmental factors and quality changes, a comprehensive assessment method for volatile gas components associated with oyster quality changes has been proposed. While traditional prediction models employ BP neural networks to adapt to various input data patterns, enhance performance through the learning of historical data, and continually optimize their efficacy, the number of hidden neuron nodes in the BP neural network algorithm can influence the learning fitting process and potentially lead to overfitting. In this study, the selection of the BP neural network model is optimized using a genetic algorithm (GA), resulting in enhanced processing capabilities for nonlinear relationships. The construction process of the GA-BP neural network model is illustrated in Figure 5.

**Step 1:** Input the stress factor parameters and the stress parameters collected, determine the number of neuronal nodes in each layer of the neural network, and classify test dataset from predictive training set;
**Step 2:** Establish relevant parameters for the genetic algorithm including the chromosome coding method, the selection operation implementation algorithm, the fitness function, and the probability of crossover and mutation operation;
**Step 3:** Generate the original population based on the neural network structure with random individual real numbers encoding the network weight and threshold information;
**Step 4:** Evaluate the adaptability of each individual in the population, inherit excellent individuals for crossover and variation into next generation, output optimal individual after several iterations;
**Step 5:** Decipher the optimal individual, allocate the optimal threshold and initial weight to the neural network, and conduct training and prediction based on the optimal weight and threshold.

## 3. Results and Discussion

### 3.1. Response of Electronic Nose to Oysters of Different Qualities

**Figure 6:**
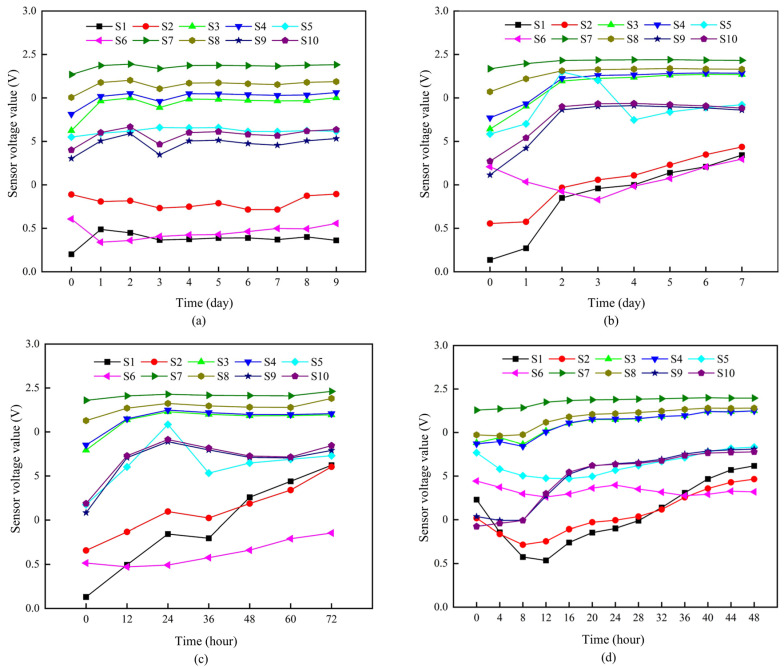
Signal response from the ten sensors of the e-nose system: (**a**) sensor output voltages of oyster samples stored at 4 °C from day 0 to day 9; (**b**) sensor output voltages of oysters samples stored at 12 °C from day 0 to day 7; (**c**) sensor output voltages of oyster samples stored at 20 °C from hour 0 to hour 72; (**d**) sensor output voltages of oyster samples stored at 28 °C from hour 0 to hour 48.

The sensor output voltages of oysters under various storage conditions are illustrated in Figure 6. Aquatic product volatiles include trimethylamine, methylmercaptan, 3-methyl-1-butanol, dimethyl disulfide, and dimethyl trisulfide [39]. When these gasses interact with the hot surface of the sensor, electron transfer occurs, leading to an increase in sensor conductance.

The conductance responses of sensor TGS2620, which is more sensitive to alcohol series volatiles, rose rapidly during the initial stage of storage. This finding aligns with the results reported by Olafsdottir et al. Additionally, sensors MQ137, TGS2600, TGS2602, and TGS2603, according to the data sheet from Figaro Co., Ltd., Osaka, Japan, demonstrated greater sensitivity to amine series volatiles [40]. An increased response was observed at the end of the storage period for amine series sensors in samples subjected to abusive temperature treatments.

**Figure 7:**
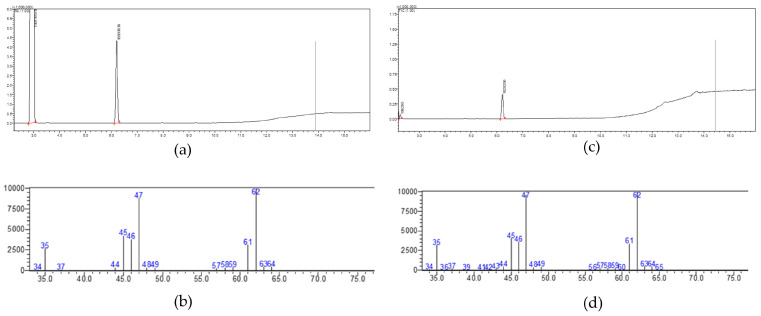
GC-MS spectra of dimethyl sulfide standard and oysters; (**a**) TIC of dimethyl sulfide standard; (**b**) MS spectrum of peak 1; (**c**) TIC of oyster sample stored at 28 °C for 48 h; (**d**) MS spectrum of peak 2.

The final phase of deterioration is associated with the appearance of sulfur, which aligns with the results from the GC-MS experiments conducted in this research. From Figure 7, it can be inferred that peak 2 from the total ion chromatogram (TIC) of the oyster sample shares the same retention indices and fragment ions (*m*/*z* 62, 61, 47, 46) as peak 1. Consequently, peak 2, which has a retention time of 6.2 min and was stored at 28 °C for 48 h, is identified as dimethyl sulfide. Furthermore, the $X_{st}$ is measured at 0.3821 ppm, the $A_{st}$ is 18,796,229, and $A_{0}$ is 1,900,211.5. Using the following formula, the concentration of dimethyl sulfide in the oyster samples ($X_{0}$) can be calculated to be 0.0386 ppm. Additionally, the aforementioned peak is the only one observed in the volatile gas TIC of oysters stored at 28 °C for 48 h. Therefore, it can be concluded that the content of sulfur compounds is likely much higher than that of other compounds, such as amines.

(1) $$X_{0}=X_{st}A_{0}/A_{st}$$

where $X_{0}$ is the dimethyl sulfide concentration of oyster samples stored at 28 °C for 48 h; $X_{st}$ is the dimethyl sulfide concentration of the standard substance; $A_{0}$ is the peak area of oyster samples stored at 28 °C; and $A_{st}$ is the peak area of the standard substance.

### 3.2. Quality Index Evaluation

**Figure 8:**
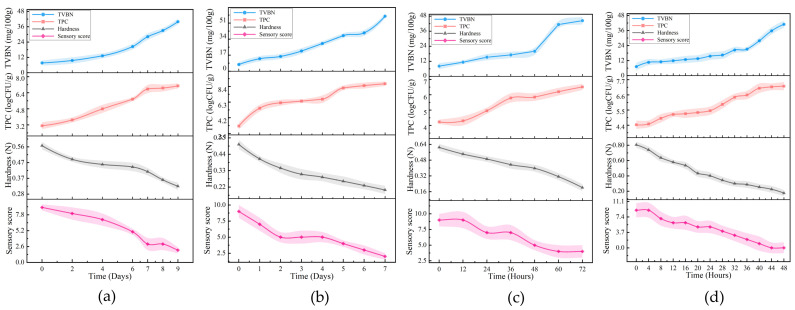
Changes in TVBN, hardness, sensory score and total number of colonies with storage time (in days and hours): (**a**) preservation at 4 °C from day 0 to day 9; (**b**) preservation at 12 °C from day 0 to day 7; (**c**) preservation at 20 °C from hour 0 to hour 72; (**d**) preservation at 28 °C from hour 0 to hour 48.

At 4 °C, the total volatile basic nitrogen (TVBN) content on day seven increased to 28.2 mg/100 g (as shown in Figure 8). At 12 °C, the TVBN content on day four rose to 29.3 mg/100 g (as illustrated in Figure 8b). In contrast, at 20 °C, the TVBN content rapidly escalated to 41.3 mg/100 g at hour 60 (i.e., day 3) (as depicted in Figure 8). Notably, under the condition of 28 °C, the TVBN content reached a level exceeding the spoilage standard of 25 mg/100 g within 36 h (as shown in Figure 8d), indicating that low-temperature storage conditions effectively delayed the spoilage process to a certain extent. Figure 8a–d illustrates the temporal changes in the total plate count (TPC) of oyster samples at different temperatures. Initially, the TPC at the four temperature conditions were 3.31 log CFU/g, 3.08 log CFU/g, 4.23 log CFU/g, and 4.11 log CFU/g, respectively, all of which were below the standard for fresh oysters (6.70 log CFU/g), indicating the excellent freshness of the samples used in the experiment. However, as the storage time increased, the microbial growth and reproduction accelerated. At 12 °C, the TPC of the oyster sample reached 8.20 log CFU/g on day five (as shown in Figure 8b), significantly exceeding the spoilage threshold for oysters (7.70 log CFU/g). This trend underscores the impact of temperature on the freshness and microbial growth of oyster samples during storage.

The experimental data were utilized to monitor variations in oyster hardness at different temperatures during storage, as depicted in panels a-d of Figure 8. It is evident that the texture parameters of oysters at various temperatures exhibited a gradual decrease with a prolonged storage time. Notably, at 28 °C, after 48 h of storage, the hardness of the oysters significantly decreased to 0.17 N. The texture properties of oyster tissue are primarily influenced by myofibrillar protein; thus, the rapid decline in texture parameters can be attributed to changes in myofibrillar protein during storage. Furthermore, this trend aligns with observations from electronic nose data, further validating our findings and analysis results. Additionally, Figure 8 presents the sensory evaluation results of oyster samples subjected to different temperature conditions across various time intervals. It was observed that, over time, the scores of the oyster samples demonstrated a gradual decline. Specifically, at 20 °C, from the 60th hour to the 72nd hour, the score of the sample decreased to below five points, indicating spoilage and rendering the quality of the oyster sample unacceptable at this stage. Similarly, at 28 °C, from hour 20 to hour 48, the sample’s rating also dropped below five, again meeting the criteria for spoilage. These observations were consistent with the results of previous textural analyses, further confirming the variation in the quality of oyster samples under specific time and temperature conditions.

### 3.3. Freshness Prediction

#### 3.3.1. Correlational Analysis

**Figure 9:**
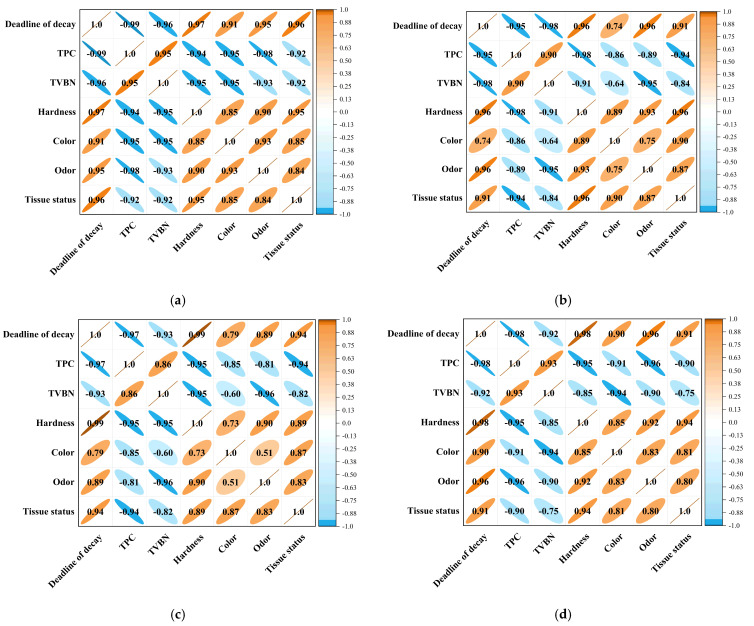
Correlation coefficients between oyster quality indicators and the deadline of decay: (**a**) preservation at 4 °C from day 0 to day 9; (**b**) preservation at 12 °C from day 0 to day 7; (**c**) preservation at 20 °C from hour 0 to hour 72; (**d**) preservation at 28 °C from hour 0 to hour 48.

Based on the correlation results of each indicator presented in Figure 9, the relationship between the quality indicators and the oyster’s shelf life can be established. The total plate count (TPC) and total volatile basic nitrogen (TVBN) exhibited a negative correlation with shelf life, whereas the other quality indicators demonstrated a positive correlation.

The correlation coefficient between the color and the decay deadline ranged from 0.74 to 0.91, indicating a relatively weak relationship across different temperatures. Consequently, color was excluded from the oyster quality prediction model. In contrast, the correlation coefficients for hardness, odor, and tissue status with the decay deadline under varying temperatures were all above 0.85, indicating a strong linear correlation. The correlation coefficients between TPC and the decay deadline ranged from 0.95 to 0.99, while those for TVBN and the decay deadline ranged from 0.92 to 0.98, both exceeding 0.95 and indicating a strong degree of correlation.

**Figure 10:**
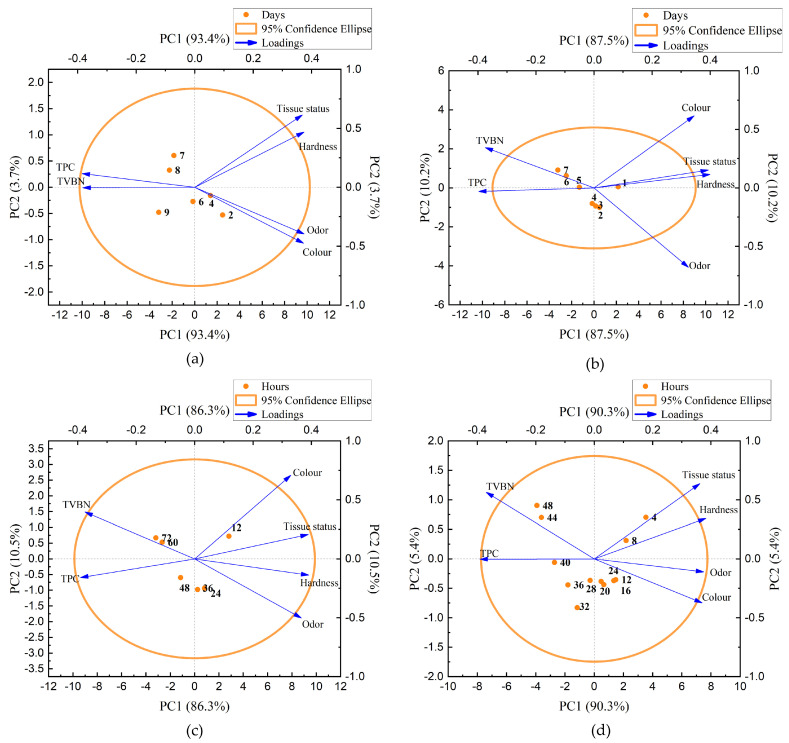
Cluster analysis: (**a**) preservation at 4 °C from day 0 to day 9; (**b**) preservation at 12 °C from day 0 to day 7; (**c**) preservation at 20 °C from hour 0 to hour 72; (**d**) preservation at 28 °C from hour 0 to hour 48.

Figure 10 illustrates the cluster analysis of the hardness, TVBN value, sensory rating score, and total number of colonies of oysters at different temperatures. These data points are categorized into distinct clusters, where objects within the same cluster exhibit high similarity, while objects in different clusters show significant differences. For two n-dimensional data points, $A=(a_{1},a_{2},\ldots ,a_{n})$ and $B=(b_{1},b_{2},\ldots ,b_{n})$, the Euclidean distance between A and B can be calculated:

(2) $$\rho \left(A,B\right)=\sqrt{\sum_{i}^{n}{\left(a_{i}-b_{i}\right)}^{2}},i=1,2,\ldots ,n$$

The orange ellipse indicates the 95% confidence interval, demonstrating the feasibility of the clustering analysis method. Utilizing a principal component analysis (PCA), the quality indices of the oysters were reduced to two, resulting in two cluster centers and ultimately leading to two comprehensive indices: PC1 and PC2. Both PC1 and PC2 accounted for over 95% of the total contribution variance, representing the majority of the information from the raw data. In Figure 10a, the projections of TVB-N and TPC on the PC1 axis were larger, indicating a greater contribution to PC1 and a more significant effect on this component. Conversely, the projections of tissue status, hardness, odor, and color on the PC2 axis were larger, suggesting a more substantial contribution to PC2 and a more significant effect on this component. In the PCA loading figure, if the angle between two variables is acute, approaching 0°, it indicates a positive relationship between them. Conversely, if the angle is obtuse, approaching 180°, it indicates a negative relationship. In Figure 10a, the angle between TVB-N and TPC was very acute, indicating a strong positive relationship between these two variables; a similar relationship was observed among tissue status, hardness, odor, and color. However, the angles between TVB-N and the other indices, such as tissue status, hardness, odor, and color, were sufficiently large, indicating a strong negative relationship between TVB-N and each of the remaining four indices, as was the case with TPC. This analysis aligns precisely with the findings presented in Figure 9a. Similar analyses can be performed for Figure 10b–d. Consequently, we categorized the six quality indices into physical and chemical categories to represent the shelf life of oysters. Among these, the physical indices, such as color and tissue status, accounted for the most significant proportion (~85%), in contrast to the lesser impact of chemical indices, such as TVB-N and odor (~10%).

#### 3.3.2. The PCA and GA-BP Array Data of the Gas Sensor

Data were collected at temperatures of 4 °C, 12 °C, 20 °C, and 28 °C, yielding 4200, 4800, 4200, and 7800 sets, respectively. The initial data were normalized to mitigate the impact of varying dimensions on the model, and dimensionality reduction was performed using principal component analysis (PCA).

PCA is a multivariate statistical method that transforms and reorients the coordinate system of the original data, aligning the origin of the processed coordinate system with that of the initial one. The new coordinates are oriented such that one axis corresponds to the direction of maximum variance in the original data, while each subsequent axis is orthogonal to those preceding it.

**Figure 11:**
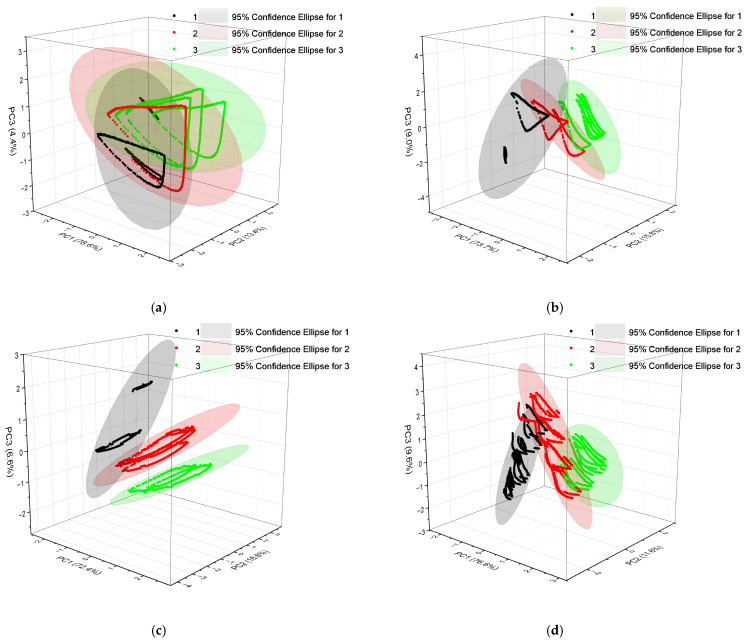
Principal component analysis (PCA) of a gas sensor array. (**a**) Preservation at 4 °C from day 0 to day 9; (**b**) preservation at 12 °C from day 0 to day 7; (**c**) preservation at 20 °C from hour 0 to hour 72; (**d**) preservation at 28 °C from hour 0 to hour 48.

As depicted in Figure 11, the cumulative contributions of the first, second, and third principal components accounted for 96.4%, 98.5%, 97.6%, and 97.8% of the original information in the dataset at temperatures of 4 degrees, 12 degrees, 20 degrees, and 28 degrees, respectively. Consequently, the original datasets corresponding to the first three principal components were utilized as input labels for GA-BP analysis, while the outcomes categorized based on the TVB-N results (with values of 1 indicating fresh, 2 representing sub-fresh, and 3 denoting decayed) served as output labels for GA-BP analysis. The experimental data pertaining to gas storage in oysters under varying temperatures were partitioned into two sets at an allocation ratio of 8:2 for training and testing purposes.

**Figure 12:**
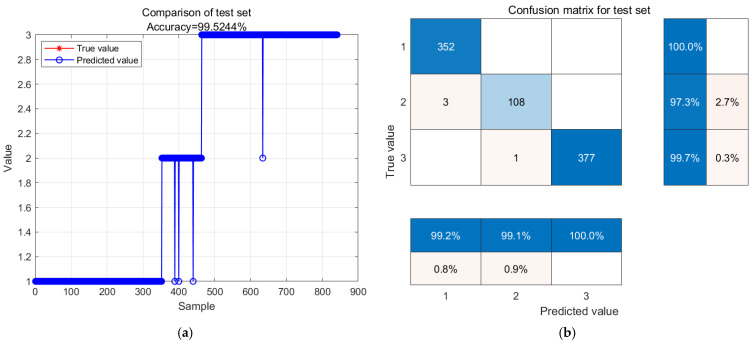
GA-BP neural network model prediction results and confusion matrix analysis stored at 4 °C. (**a**) GA-BP neural network model prediction results; (**b**) confusion matrix analysis.

The predicted results of the gas quality grade model at 4 degrees Celsius are presented in Figure 12. Figure 12a illustrates the comparison between the actual and predicted values, with red representing the actual values and blue denoting the predictions. The test model achieved an accuracy of 99.5244%, with all the prediction errors occurring at the sub-fresh and spoiled levels, while successfully predicting all samples at the fresh level. In Figure 12b, among the 352 fresh level samples, no samples were predicted as other levels, with an accuracy of 100.0%. Out of 111 sub fresh samples, three were incorrectly predicted as fresh with an accuracy rate of 97.3%. Out of the 378 samples with decay levels, one was incorrectly predicted as sub fresh level with an accuracy rate of 99.7%.

**Figure 13:**
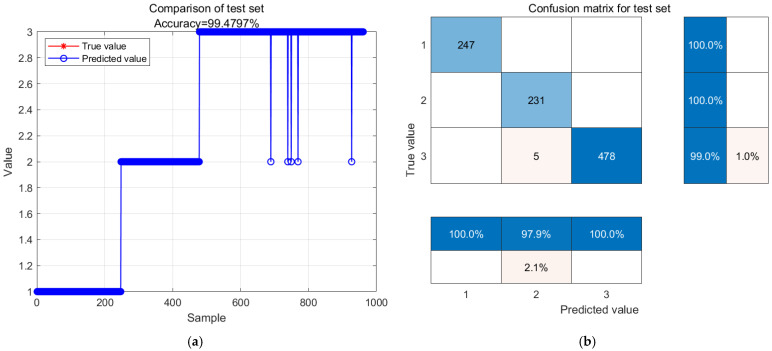
GA-BP neural network model prediction results and confusion matrix analysis stored at 12 °C. (**a**) GA-BP neural network model prediction results; (**b**) confusion matrix analysis.

Figure 13 shows the predicted results at a temperature of 12 degrees Celsius. In Figure 13a, the comparison between actual values and predicted values is shown, with red representing actual values and blue representing predicted values. The accuracy of the test model is 99.4797%, and all the prediction errors occur at the decay level. In Figure 13b, all 247 of the fresh samples and 231 of the sub-fresh samples were predicted correctly. Out of the 483 spoiled samples, only five were predicted as sub fresh with an accuracy rate of 99.0%.

**Figure 14:**
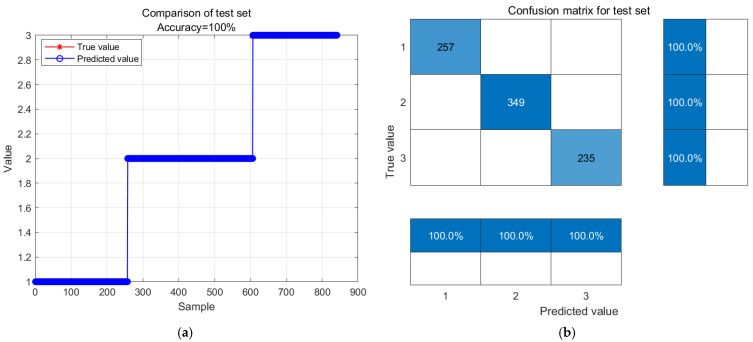
GA-BP neural network model prediction results and confusion matrix analysis stored at 20 °C. (**a**) GA-BP neural network model prediction results; (**b**) confusion matrix analysis.

The predicted results at 20 degrees Celsius are shown in Figure 14. In Figure 14a, the actual values of the model are compared with the predicted values, where red represents the actual values and blue represents the predicted values. The accuracy of the test model is 100.0%. 257 fresh level samples, 349 sub-fresh level samples, and 235 spoiled level samples were all predicted correctly. This proves the excellent performance of the model.

**Figure 15:**
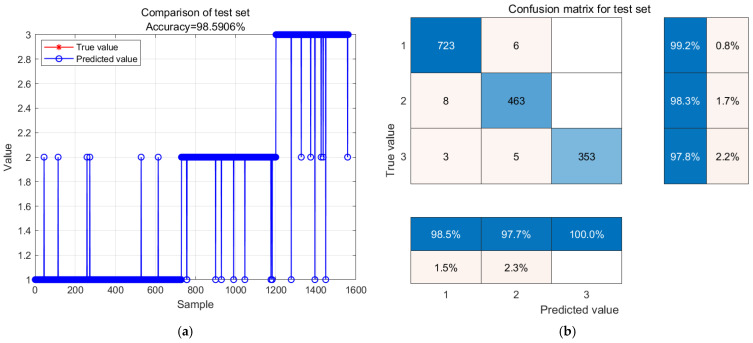
GA-BP neural network model prediction results and confusion matrix analysis stored at 28 °C. (**a**) GA-BP neural network model prediction results; (**b**) confusion matrix analysis.

Figure 15 shows the predicted results at a temperature of 28 degrees Celsius. In Figure 15a, the comparison between the actual and predicted values of the model is shown, with red representing the actual values and blue representing the predicted values. The accuracy of the test model is 98.5906%, and the prediction error mainly comes from the confusion between sub fresh and fresh, as well as between rot and the other two grades. In Figure 15b, out of the 729 samples classified as fresh, six were incorrectly predicted as sub-fresh with an accuracy of 99.2%. Out of the 471 sub fresh samples, eight were incorrectly predicted as fresh with an accuracy rate of 98.3%. Among the 361 samples at the spoiled level, five were incorrectly predicted as sub-fresh, and three were incorrectly predicted as fresh, with an accuracy rate of 97.8%.

In conclusion, the accuracy of oyster gas quality grading at various temperatures exceeds 95%, demonstrating that the classification and prediction of oyster quality grade can be accomplished through the integrated model of oyster gas quality grading.

### 3.4. System Evaluation

Table 4 presents a comprehensive comparison between the traditional system and optimized system in detail. In contrast to the traditional system, the proposed prediction system integrates an array of gas sensors into intelligent IoT monitoring, achieving higher accuracy while enabling multi-parameter monitoring. Additionally, the traditional evaluation method fails to consider various quality indicators and sensory evaluation on quality under any circumstances. The improved system conducts data statistical analysis on collected different signals (volatile basic nitrogen content, total number of colonies, texture parameters and sensory evaluation indexes, etc.) as quality indices for fresh oysters. Through analyzing the collected data, the sensor array real-time dynamic monitoring system has demonstrated outstanding performance in evaluating oyster freshness.

In this study, relevant personnel are invited to participate in the system performance analysis of the sensor array, and an optimization strategy is proposed to enhance the practicality and applicability of the established monitoring system. The system evaluation team consists of staff, managers, research group members, and experts from cold chain logistics-related enterprises. Despite optimization efforts, the device still exhibits high battery power consumption and extensive calculation requirements, leading to poor sustainability and low equipment efficiency. It is recommended that future devices reduce the number of single gas sensors and minimize equipment volume. Additionally, other textural analysis parameters (such as springiness, cohesiveness, gumminess, and chewiness) can be considered to bolster quality evaluation credibility. Furthermore, calibrating the gas sensor array with GC-MS can improve the accuracy of the quality prediction models.

### 3.5. Discussion

As shown in Table 5, Kuuliala et al. employed a MOS sensor array and Luzuriaga used conductive polymer sensors to detect the oyster freshness. Although these models demonstrated reasonable accuracy in detecting volatile organic compounds associated with seafood spoilage, their prediction accuracy remained limited by the sensitivity and specificity of the sensors to the volatile compounds in different storage conditions. For example, the MOS sensor array in Kuuliala’s study reached an accuracy of 85%, but it was susceptible to interference from environmental factors such as temperature fluctuations. However, the proposed method improved upon these limitations by integrating a more advanced MOS sensor array with a GA-BP (genetic algorithm–back propagation) neural network model. This allowed for better adaptation to the complex and variable conditions associated with oyster freshness, particularly through enhanced gas recognition and multi-parameter sensing capabilities. The application of machine learning in this study led to significantly higher prediction accuracy (97.9%) across a broader range of temperatures (4–28 °C), demonstrating the robustness and adaptability of the system in real-world conditions. In addition, compared to the previous study, the proposed one significantly expands the detection ranges, including trimethylamine, ammonia, and disulfides, which are critical markers of spoilage in oysters. This broad detection range enables a more comprehensive assessment of oyster freshness, contributing to the improved accuracy of freshness prediction models. Furthermore, the current study includes a more comprehensive set of freshness indicators, including TVBN, total plate count (TPC), texture, sensory analysis, and hardness. This multi-parameter approach provides a more holistic understanding of oyster freshness, ensuring that subtle changes in both physical and microbial parameters are captured. The integration of these indicators with the GA-BP neural network further enhances the prediction model’s ability to accurately classify the freshness of oysters. Despite the advancements demonstrated in this study, some challenges remain. For instance, while the GA-BP neural network model significantly improved prediction accuracy, the model requires further optimization to ensure it performs consistently across different environmental conditions and oyster varieties. Moreover, the current study focuses on laboratory settings, and further validation is necessary in real-world commercial environments to ensure the system’s scalability and robustness. Future research should also explore the integration of other machine learning algorithms and advanced sensor technologies, such as deep learning and hyperspectral imaging, to further enhance the accuracy and applicability of freshness prediction models.

## 4. Conclusions

In this study, we proposed a machine learning-based model for predicting the freshness of oysters, which enables the assessment of oyster quality at various storage temperatures. This model incorporates electronic nose technology that facilitates the real-time and intelligent sensing of volatile gasses released by oysters, along with multiple quality indicators, thus constituting a comprehensive system for evaluating oyster quality.

1. We designed and established a real-time dynamic monitoring system for the cold chain transportation of freshly captured oysters;
2. We deduced the factors influencing the quality of fresh oysters based on their logistics process and quality change mechanisms, while also discussing the feasibility and correlation between different signals as indicators of oyster quality;
3. We developed a neural network-based coupling model that integrates gas sensor information with oyster quality and verified its effectiveness. The results demonstrated that the prediction accuracy for classifying the gas-quality grade of oysters at different temperatures exceeded 95%, indicating that the classification and prediction of oyster quality grades can be effectively achieved through this coupling model.

## Figures and Tables

**Table 1:** The sensor specifications utilized in the Oyster Nose model.

S. No	Sensor Types	Volatile Compounds	Detection Range (ppm)
S1.	TGS2602	Ammonia, hydrogen sulfide, and toluene	1~30
S2.	TGS2603	Amine series, hydrogen sulfide, etc.	1~10
S3.	TGS2612	Methane, propane, isobutane, etc.	500~10,000
S4.	TGS2630	Refrigerant gas	1000~10,000
S5.	MQ137	Ammonia and amine compounds	5~500
S6.	MQ135	Ammonia gas, sulfide, benzene series vapor	10~1000
S7.	TGS2611	Ethanol, hydrogen, isobutane, methane	500~10,000
S8.	TGS2610	Ethanol, hydrogen, methane, isobutane/propane	500~10,000
S9.	TGS2620	Organic solvents, alcohol, etc.	50~5000
S10.	TGS2600	Carbon monoxide, hydrogen	1~30

**Table 2:** Experimental grouping method.

Monitoring Items	4 °C		12 °C		20 °C		28 °C	
	Test Node	Duration	Test Node	Duration	Test Node	Duration	Test Node	Duration
TVBN	Days 0, 2, 4, 7, 8, 9	Day 10	Every day	7 days	Every 12 h	72 h	Every 4 h	48 h
Microbial analysis								
Sensory evaluation								
Texture analysis								

**Table 3:** Sensory evaluation criteria of oyster meat.

Sensory Score	Color (10 Points)	Odor (10 Points)	Tissue Status (10 Points)
8–10	Milky or cream white and shiny	It smells normal and has no odor	Good elasticity, quick rebound after pressing, firm flesh
5–7	White with an eggy yellowish color, slightly dull in color	Slightly fishy	Elasticity is general, after pressing it cannot be fully restored to its original state, the meat is firmer
0–4	Yellowish and noticeably dull	Distinctly fishy	Poor elasticity, does not return to its original state after pressing, and the flesh is soft or tends to be mushy

**Table 4:** Comprehensive comparison of the traditional model and the proposed model.

Model Performance	Sensors Performance and Environmental Parameters Evaluation						Quality Analysis and Evaluation				Prediction Model Evaluation
	Monitoring Parameters	Temperature	Humidity	Amine Series	Sulfide Series	Alkane Series	4 °C	12 °C	20 °C	28 °C	
Previous monitoring method	Temperature and Humidity	Range: −40–120 °C Accuracy: ±0.4 °C	Range: 0–100%RH Accuracy: ±3% RH	None	None	None	None				None
Improved model	Temperature Humidity Amine series Sulfide series Alkane series	Range: −40–70 °C Accuracy: ±0.2 °C	Range: 0–100%RH Accuracy: ±1% RH	Range: 5–500 ppm 0–100 ppm 1–30 ppm 0–0.5 ppm	Range: 1–30 ppm 0–0.5 ppm	Range: 500–10,000 ppm 0.1–0.25 ppm	TVBN ≤ 25	TVBN ≤ 25	TVBN ≤ 25	TVBN ≤ 25	The accuracy at different temperatures is all greater than 95%
							TPC ≤ 7.70	TPC ≤ 7.70	TPC ≤ 7.70	TPC ≤ 7.70	
							Sensory score ≥ 5	Sensory score ≥ 5	Sensory score ≥ 5	Sensory score ≥ 5	
Advantages	Multiple critical parameters monitoring	Trace monitor of multiple gas components Better traceability and accuracy of temperature and humidity Real-time, online monitoring and non-destructive Validation by GC-MS					Different quality evaluation standard under different temperature detailed and comprehensive quality evaluation				Predict accurately and effectively without contamination and contact
Suggestions	More critical parameters	Develop smaller size gas sensor array with fewer numbers of single sensors for oysters’ storage					Only hardness was measured; textural analysis (springiness, cohesiveness, gumminess, and chewiness) still needs to be augmented and improved				Calibrate the gas sensor array with GC-MS to improve accuracy

**Table 5:** Comprehensive comparison of the traditional electronic nose technology in oyster freshness detection and the proposed method.

Parameter	Kuuliala et al. (2018) [8]	Luzuriaga et al. (2008) [9]	Proposed Study
Electronic Nose Model	MOS sensor array	Conductive polymer sensor array	MOS sensor array with GA-BP neural network
Detected Volatile Compounds	Trimethylamine, ammonia, hydrogen sulfide	Carbon dioxide, ethanol	Trimethylamine, ammonia, disulfides
Storage Temperature Range (°C)	4–8 °C	4 °C	4–28 °C
Prediction Accuracy (%)	85%	99.2%	97.9%
Freshness Indicators	TVBN, Microbial Analysis, Sensory Analysis	Sensory Analysis	TVBN, TPC, Texture, Sensory, Hardness
Machine Learning Applied	None	None	GA-BP Neural Network Model
Sampling Frequency	Once per day	Once every 2 days	Hourly
Experiment Duration	7 days	48 h	9 days
Key Findings	High repeatability and reliability	High accuracy sensory evaluation	Integrated real-time multi-parameter monitoring and machine learning predictions

## Data Availability

The data presented in this study are available on request from the corresponding author.
